# Does probiotic consumption reduce antibiotic utilization for common acute infections? A systematic review and meta-analysis

**DOI:** 10.1093/eurpub/cky185

**Published:** 2018-11-14

**Authors:** Sarah King, Daniel Tancredi, Irene Lenoir-Wijnkoop, Kelsie Gould, Hailey Vann, Grant Connors, Mary Ellen Sanders, Jeffrey A Linder, Andi L Shane, Dan Merenstein

**Affiliations:** 1 Cambridge, UK; 2 University of California, Davis, CA, USA; 3 University of Utrecht, Utrecht, The Netherlands; 4 Georgetown University, Washington, DC, USA; 5 International Scientific Association for Probiotics and Prebiotics; 6 Northwestern University Feinberg School of Medicine, Chicago, IL, USA; 7 Emory University School of Medicine, Atlanta, GA, USA

## Abstract

**Background:**

Overall reduction of antibiotic use is a widely adopted public health goal. Given evidence that consuming probiotics reduce the incidence, duration and/or severity of certain types of common acute infections, we hypothesized that probiotics are associated with reduced antibiotic use. This systematic review of randomized controlled trials (RCTs) assessed the impact of probiotic supplementation (any strain, dose or duration), compared to placebo, on antibiotic utilization for common, acute infections in otherwise healthy people of all ages.

**Methods:**

We searched 13 electronic databases including MEDLINE, Embase and CENTRAL from inception to 17th January 2017. Backward and forward citation searches were also conducted. Two reviewers independently selected studies for inclusion and extracted study data. We assessed risk of bias for individual studies using criteria adapted from the Centre for Reviews and Dissemination, and the quality of evidence for each outcome was assessed using the GRADE system. Studies that evaluated similar outcomes were pooled statistically in meta-analyses using a random-effects model.

**Results:**

We screened 1533 citations, and of these, 17 RCTs met our predefined inclusion criteria. All 17 were conducted in infants and/or children with a primary aim of preventing acute respiratory tract infections, acute lower digestive tract infections or acute otitis media. Included studies used 13 probiotic formulations, all comprising single or combination *Lactobacillus* and *Bifidobacterium* delivered in a range of food or supplement products. Mean duration of probiotic supplementation ranged from 4 days to 9 months. Trial quality was variable. Meta-analysis demonstrated that infants and children who received probiotics to prevent acute illnesses had a lower risk of being prescribed antibiotics, relative to those who received placebo (Pooled Relative Risk = 0.71, 95% CI: 0.54–0.94). When restricted to five studies with a low risk of bias, the pooled relative risk was 0.46 (95% CI: 0.23–0.97). Significant statistical heterogeneity was present in effect size estimates, which appeared to be due to one trial which could partly be considered as an outlier.

**Conclusions:**

Probiotics, provided to reduce the risk for common acute infections, may be associated with reduced antibiotic use in infants and children. Additional well-designed studies are needed to substantiate these findings in children and explore similar findings in other population groups.

## Introduction

The threat of antibiotic resistant pathogens has led many public health organizations around the globe to work toward improving the appropriate use of antibiotics, defined by WHO as “the cost-effective use of antimicrobials which maximizes clinical therapeutic effect while minimizing both drug-related toxicity and the development of antimicrobial resistance”.^1^ According to the CDC, there are about two million cases of antibiotic-resistant infections yearly in USA, resulting in 23 000 deaths.^2^ One means to reduce antibiotic use is to avoid prescribing them for viral illnesses such as colds, influenza and other acute respiratory infections, where inappropriate antibiotic use is most common.^3–5^ Another means to reduce antibiotic use is to help reduce the frequency and the duration of symptoms; if fewer people get ill, fewer people may be prescribed antibiotics—and if people are symptomatic for shorter periods of time, they may be less likely to be prescribed antibiotics, or to request antibiotic treatment in order to alleviate prolonged symptoms.

Probiotics are live microorganisms that, when administered in adequate amounts, confer a health benefit on the host.^6^ Probiotics are used to prevent and treat many gastrointestinal maladies.^7^ Lately, targets for probiotic benefits have extended beyond the gut.^8^ Probiotic effects are mediated by various and sometimes strain-specific mechanisms, including the strengthening of gut barrier structure and function; interactions with immune system components; production of short-chain fatty acids in the gut, and other direct and indirect influences on the stability, expression and composition of host microbes.^9^

Evidence suggests that probiotic supplementation reduces episodes of common infectious diseases including respiratory tract infections^10^ and diarrhea.^11^ In addition, probiotic supplementation reduces the duration of symptoms in otherwise healthy children and adults with common acute respiratory conditions.^12^ By decreasing the incidence and severity of common acute infections, probiotic supplementation could be associated with decreased antibiotic use.

To our knowledge, the relationship between probiotic use and antibiotic use has not been systematically reviewed. The objective of this systematic review is to explore whether or not antibiotic prescriptions are reduced in the target populations of studies that investigated probiotics to reduce the risk for common acute illnesses. We hypothesize that because probiotics help to reduce the incidence or duration of certain types of common acute infections, probiotic supplementation could be associated with reduced consumption of antibiotics. If so, probiotic supplementation could be an evidence-based strategy to reduce the need for antibiotics and therefore could contribute to managing the emergence of antibiotic resistance.^13^

## Methods

### Inclusion/exclusion criteria

In this systematic review, we included randomized controlled trials (RCTs) of any duration in which probiotic supplementation was provided to reduce the risk for common acute infections in healthy people of all ages (i.e. infants, children, adolescents and/or adults, including elderly/institutionalized elderly). Eligible study infections included acute respiratory tract infections (i.e. colds, influenza, sinusitis, pharyngitis, acute bronchitis, pneumonia), acute otitis media and acute lower digestive tract infections (diarrhea), but *not* antibiotic-associated gastrointestinal symptoms. We included conditions where symptoms were self-reported by a participant (or a parent in the case of infants and children), or where upper or lower tract respiratory symptoms were medically attended for 14 days or less.

The included studies also had to report a measurement of antibiotic prescription, receipt or consumption (e.g. mean number, percentage, the number or percentage of participants with more than one antibiotic prescription, antibiotic purchases, etc.) within the intervention period or within two weeks after the intervention period, either as a primary or secondary outcome.

We included studies that assessed any orally consumed probiotic strain, either alone or in combination with another probiotic strain, and that compared probiotics with a placebo or with no treatment. Studies where probiotics were combined with other potentially functional ingredients or therapies (e.g. prebiotics, vitamins, herbal extracts, immunostimulants, drugs such as antihistamines, or non-steroidal anti-inflammatory drugs) were eligible only if the comparator also included these other interventions as well, so that the net effect of probiotic supplementation could be assessed. We included studies conducted in any country and in any setting, clinical or non-clinical.

### Search strategy

To identify relevant trials, we searched MEDLINE, Embase, CINAHL, the Cochrane Database of Systematic Reviews (CDSR), the Cochrane Central Register of Controlled Trials (CENTRAL), the Database of Abstracts of Reviews of Effects (DARE), NIHR Health Technology Assessment (HTA) database, NICE Technology appraisals, Science Citation Index (SCI) and Conference Proceedings Citation Index. We searched for grey literature using OAISTER, OpenGrey and NYAM Grey Literature Report, from inception to 17 January 2017. Search terms included ‘probiotics’, ‘antibiotics’ and ‘antimicrobials’. The full list of search terms is presented in Supplementary Appendix A. Although we did not impose any language or date restrictions, no non-English papers were identified that met the inclusion criteria.

We also applied additional evidence-finding techniques, including checking references cited within the included papers and relevant systematic reviews (i.e. backward citation searching); searching for additional studies by the first (or primary) authors of relevant studies; and forward citation searches to identify subsequent studies/publications which had cited the included studies.

### Screening and data extraction

Two independent reviewers (SK and HV) screened titles and abstracts identified through the searches against the pre-defined inclusion/exclusion criteria. Full papers were obtained of any records deemed to be potentially relevant during the first screening phase, and these were also screened independently by two reviewers (SK and KG). We resolved any discrepancies through discussion.

For each eligible study, data on study population characteristics and results were extracted by one reviewer and checked by a second reviewer (SK and KG). In addition, the results were independently extracted by a third reviewer (HV) and the two extraction forms compared.

### Risk of bias assessment

We assessed risk of bias of the RCTs using criteria adapted from the Centre for Reviews and Dissemination.^14^ One reviewer applied this criteria (SK) which was then checked by a second reviewer (KG). A summary assessment for each study was conducted following Cochrane guidance.^15^ We considered that the key quality criteria involved protections against selection and attrition biases: adequate randomization and allocation concealment and adequate analysis of data from enrolled participants. Individual studies were considered to have a ‘low’ risk of bias if all three key criteria were adequately met, and a ‘high’ risk of bias if one or more of these criteria were not adequately met. As antibiotic use is an objective outcome and not likely to be affected by a lack of blinding of the investigators or participants, it was not considered to be key quality criterion.

For each outcome evaluated in our systematic review, the group of studies that contributed data to this outcome was assessed to be of ‘high’, ‘moderate’, ‘low’ or ‘very low’ quality (as a body of evidence) using the GRADE system.^15^ This system involves up or down-grading the evidence based on risk of bias of the studies that evaluated the outcome, as well as imprecision, inconsistency, indirectness and publication bias.

When outcome data or information needed to evaluate quality criteria were unclear in the publications, we attempted to contact study authors for further information.

### Data synthesis

To inform judgments about the comparability of studies prior to combining their results via meta-analysis, study attributes were described and compared with regard to the following: population characteristics (e.g. age group, infection type and patient eligibility status relative to the study’s therapeutic objective [prevention vs. treatment]); intervention (level of shared probiotic taxonomic classification [e.g. strain, species, genera]) and outcomes (domain and type of measurement [e.g. duration of intervention, etc.]).

We conducted meta-analyses in RevMan Version 5.3. Copenhagen: The Nordic Cochrane Centre, The Cochrane Collaboration, 2014. using a random effects model. Means and standard deviations were collected for continuous outcomes and used to estimate study-specific and pooled mean differences with 95% confidence intervals (CIs). Numerators and denominators were collected for dichotomous outcomes, with Mantel-Haenszel (M-H) risk ratios (RRs) and 95% CIs used to summarize effect sizes. When a study evaluated more than one probiotic intervention arm, we combined the data from those arms to create one larger probiotic intervention arm.^16^^,^^17^ In order to include any cluster randomized trials into the meta-analyses, we adjusted the data as described in the Cochrane Handbook.^15^ To accommodate the varying levels of baseline risk for patients, numbers needed to treat (NNT) estimates can be derived from the estimated RR, using the algebraic relationship NNT = 1/ [(1−RR)*B], where *B* is a hypothesized level of baseline risk.

Heterogeneity across the primary studies in effect size and 95% confidence interval estimates was visualized using forest plots (to visualize point and 95% confidence intervals for individual treatment effect estimates), quantified using the *I*^2^ statistic, and assessed for statistical significance using the chi-squared test for heterogeneity (significance set at *P* < 0.05). Following guidelines suggested by the Cochrane Collaboration, we interpreted an *I*^2^ point estimate of <40% as being potentially not important, 30–60% as possibly representing moderate heterogeneity, 50–90% as possibly representing substantial heterogeneity, and 75–100% as possibly representing considerable heterogeneity.^15^

In the systematic review protocol, we had planned to conduct subgroup analyses by grouping studies by probiotic taxonomic classification, dose, study duration (i.e. risk-reduction studies with an intervention less than one month or longer than one month), setting (i.e. nurseries, schools, retirement homes), studies that aim to prevent or treat acute infections, and study country. We could not do these planned analyses as substantial groupings that would enable statistical comparisons were not possible. This was due to (a) a high degree of variability among the studies (for taxonomic classification, dose and study country), (b) no/little variation between the studies (for study duration, prevention vs. treatment studies) or (c) a lack of information in some of the included studies to facilitate comparisons (setting). In sensitivity analyses, we repeated the meta-analysis after omitting lower quality studies.

## Results

We assessed a total of 1533 citations for inclusion based on titles and abstracts. Of these, 88 records were considered potentially relevant and full papers were retrieved for further assessment. After screening these full papers, 17 RCTs met the inclusion criteria and were included in this systematic review.^11^^,^^16–31^ Trial locations were widely dispersed and included Chile (one trial), China (one trial), Croatia (two trials), Finland (five trials), Israel (one trial), Mexico (one trial), Sweden (one trial), Thailand (one trial), the UK (one trial), the Ukraine (one trial) and the USA (two trials).

All of the included RCTs were conducted in infants and/or children. Only one of the trials was a cluster- RCT.^26^ Among studies where setting was explicitly reported, nine were conducted in children who were attending out of home child care centres,^16–18^^,^^20^^,^^22^^,^^23^^,^^25^^,^^26^^,^^29^ one was conducted among attendees of a public school,^28^ and one was conducted in children who were hospitalized and who were at risk of developing acute gastrointestinal or respiratory infections.^24^ For this last study, the included children were reported to have been hospitalized for non-infectious gastrointestinal disorders, genetic disorders, cardiac disorders, urinary tract disorders, neurologic disorders, non-infectious pulmonary and immunologic disorders and intoxications.

The majority (11) of studies assessed the impact of probiotics on respiratory and gastrointestinal tract infections.^16^^,^^17^^,^^20^^,^^22–27^^,^^30^^,^^31^ Four studies focused on respiratory tract infections (RTI),^18^^,^^19^^,^^21^^,^^28^ and of the remaining two studies, one assessed ‘common’ conditions including respiratory and gastrointestinal tract infections,^11^ and the other assessed ‘fever, diarrhea or other illnesses’.^29^ Thirteen different probiotic interventions were assessed, although all involved individual or combinations of *Lactobacillus* and *Bifidobacterium* strains (details of the preparations are presented in Supplementary table S1). The methods of delivery also varied, and included dry powder mixed with formula, expressed breastmilk, milk or milk products, water or juice, in a cereal, or given within in a commercially pre-prepared yogurt drink, or as oil drops, or tablets in a slow-release pacifier. The intervention durations ranged from a median of 4 days to 9 months across the included trials, although the duration of probiotic in the majority of studies (15) was greater than one month. All of the trials sought to reduce the risk for infection rather than treat an acute or chronic illness. An overview of the study characteristics is presented in Supplementary table S1.

Three different outcomes relating to antibiotic use were described in the included studies: the percentage of participants who were prescribed antibiotics during the probiotic intervention period, mean or median number of antibiotic prescriptions received by subjects, and ‘mean number of days’ of antibiotic use. We note, however, that the study authors did not always distinguish between antibiotic prescriptions and consumption. A measure of antibiotic use was a primary outcome in four studies^16^^,^^17^^,^^22^^,^^25^ and a secondary outcome in nine studies.^18–21^^,^^26–29^^,^^31^ It was not explicitly stated whether it was a primary or secondary outcome in an additional four studies.^11^^,^^23^^,^^24^^,^^30^

### Percentage of participants prescribed antibiotics

**Figure 1:**
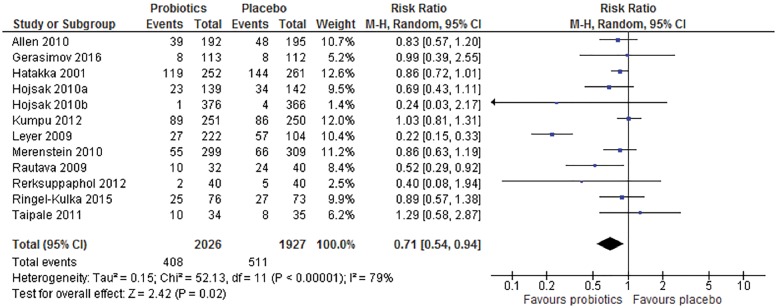
Percentage of infants/children who were prescribed antibiotics by treatment group

Of the 17 included RCTs in our systematic review, 12 reported the number of infants/children prescribed antibiotics in the intervention and control groups. In a meta-analysis, the overall relative risk of being prescribed antibiotics in infants or children who received probiotics (RR) compared to those who did not was 0.71 (95% CI: 0.54–0.94; *n* = 3953; *P* = 0.02; figure 1). For patients with a hypothetical baseline risk for receiving an antibiotic prescription of 25%, the number needed to treat to avoid one prescription would be 13.8 (95% CI: 8.7–66.7).

There was a high level of statistical heterogeneity among the studies (Tau^2^=0.15, Chi^2^*P* values=*P* < 0.001, *I*^2^=79%). When we omitted one statistical outlier (Leyer et al. 2009)^16^ from the analysis, the amount of statistical heterogeneity became negligible (Tau^2^ = 0.00; Chi^2^*P* values = 0.51; *I*^2^=0%) and the pooled RR of antibiotic use was 0.87 (95% CI: 0.78–0.97; *n* = 3627; *P* = 0.01).

In a sensitivity analysis, we removed studies considered to have a high risk of bias from the meta-analysis^11^^,^^19^^,^^22^^,^^25^^,^^27^^,^^29^^,^^30^ leaving five studies.^16^^,^^23^^,^^24^^,^^26^^,^^28^ In this analysis, children who took probiotics were less likely to receive antibiotics (RR 0.47 [95% CI: 0.23–0.97], *n* = 2067, *P* = 0.04), but again there was a high level of statistical heterogeneity among the studies (Tau^2^=0.48; Chi^2^*P* values=*P* < 0.001; *I*^2^=86%), which could be eliminated by the exclusion of one statistical outlier (Leyer et al., 2009)^16^ from the analysis. After this exclusion, the protective effect of probiotic supplementation on antibiotic receipt in these four-remaining low risk-of-bias studies is similar as that for the primary meta-analysis but becomes statistically non-significant (RR 0.78 [95% CI: 0.60–1.01], *n* = 1711, *P* = 0.06, Tau^2^=0.00; Chi^2^*P* values = 0.49; *I*^2^=0%).

As the Merenstein et al. (2010) trial was a cluster-RCT, where participants within each household were randomized as a unit, we adjusted the data to account for a reported design effect of 1.05.

### Mean or median number of antibiotic prescriptions

Two studies presented data on the mean or median number of antibiotic prescriptions given to trial participants,^17^^,^^21^ but not enough data were reported in one of the papers to calculate an effect size, so pooling of the data from both studies via meta-analysis could not be done. In this study, the authors reported that the median number of antibiotic prescriptions received was 1 (25th to 75th percentiles: 1–3) in the probiotic group (*n* = 135) and 1 (0–2) in the placebo group (*n* = 134).^21^ Sufficient data were reported in the other paper^17^ to calculate an effect size and confidence intervals. The results revealed a statistically non-significant difference between the average number of prescriptions given to those infants and children who took probiotics compared with those who received placebo (MD -0.05 [95% CI: −0.16 to 0.06], *n* = 201, *P* = 0.36). However, the sample size was small and the confidence intervals were wide. In addition, this study did not report the percentage of children in each arm that received an antibiotic prescription during the study period, so that it is unclear if there were differences between the arms which may have confounded the results.

### Mean number of days of antibiotic use

**Figure 2:**
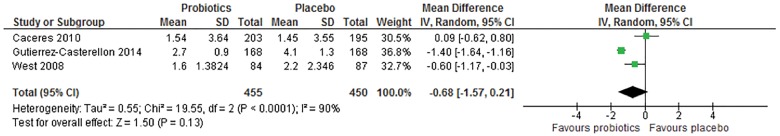
Mean number of days of antibiotic use

Three studies reported on the mean number of days that antibiotics were used.^18^^,^^20^^,^^31^ Meta-analysis did not demonstrate a significant difference in days of antibiotic use between infants and children who did and did not receive probiotics (MD -0.68 days [95% CI: −1.57 to 0.21], *n* = 905; *P* = 0.13) (figure 2). None of these studies reported if the patients took the full prescribed course or what the prescription lengths were, so it is not clear if this data reflects prescription length or consumption.

### Risk of bias and overall quality of evidence

Five of the included studies were considered to have a low risk of bias,^16^^,^^20^^,^^23^^,^^24^^,^^26^ and eight were considered to have a high risk of bias,^11^^,^^19^^,^^21^^,^^22^^,^^25^^,^^27^^,^^29^^,^^30^ primarily because not all of the infants or children who were randomized into each trial were included in the study analyses, as would be required for a strict intention-to-treat analysis. Four studies were considered to have an unclear risk of bias due to a lack of reporting on the methods of randomization sequence and/or allocation concealment.^17^^,^^18^^,^^28^^,^^31^ An overview of the risk of bias for each trial is presented in Supplementary table S2.

The quality of evidence that contributed to each of the three outcomes reported (i.e. the percentage of participants taking antibiotics during the treatment period; mean or median number of antibiotic prescriptions received; and mean number of days with antibiotic) was considered to be low using the GRADE system.

## Discussion

This systematic review aimed to assess the impact of probiotic supplementation (any strain, dose or duration), compared to placebo, on antibiotic utilization for common acute infections in otherwise healthy people of all ages. The most commonly reported outcome was the incidence of infants and children who received an antibiotic while consuming probiotics to reduce the risk for acute respiratory and gastrointestinal infections. The results from our meta-analysis of 12 studies show a 29% relative risk reduction for being prescribed antibiotics in people who consumed probiotics, while the 95% CI indicates that the true relative risk reduction could plausibly be as low as 6% or as high as 46% (pooled relative risk = 0.71, 95% CI: 0.54–0.94). The overall quality of the evidence for this outcome was, however, low. A sensitivity analysis which included only those studies with a low risk of bias (*n* = 5) also found a significant effect in favour of probiotics (pooled relative risk = 0.47, 95% CI: 0.23–0.97).

In both of our analyses, a high level of statistical heterogeneity was observed, but this was due to one statistical outlier.^16^ It is not clear why the trial results in this study were outlying, as it is similar to the others in terms of study population, intervention and duration, except that it was the only study conducted in China, where differences in environment, diet, study conduct or other factors may have contributed to the unusually strong effects observed. It could also reflect different prescribing practices and policies in the study country compared to others, but this remains unclear (Leyer pers. comm.).

The data on ‘mean or median number of prescriptions’ or ‘mean number of days of antibiotic use’ were not very informative for our purposes, because few studies reported these outcomes and details were not well described. For example, the durations of prescribed antibiotic courses were not reported in those studies that evaluated the average number of days of antibiotic use to infants and children who did and did not receive probiotics.

There are some limitations to our main finding. The majority of the trials did not explicitly state whether or not the antibiotics prescribed were for the acute infections evaluated within each study, if they were also prescribed for other reasons, or if subjects were compliant with the prescribed course of antibiotic. Therefore, although these randomized studies show a difference in rates of antibiotic prescriptions, it is not clear if this represents a causal link between probiotic use, decreased incidence and severity of infection, and reduced antibiotic use. In addition, only four of the 17 trials included in this review, evaluated antibiotic use as a primary outcome, so that many of the results may be considered as chance observations. Furthermore, all studies that met our eligibility criteria were conducted in infants and children. Although there are studies of probiotics for acute common respiratory infections in adults,^32–40^ none reported antibiotic use as an outcome, so we are unable to extrapolate our results beyond infants and children.

Probiotic consumption may be associated with reduced antibiotic prescribing for several reasons. First, probiotics have been shown to reduce the risk for common illnesses.^10^ If fewer common infections occur, there are fewer physician visits and therefore, fewer opportunities for antibiotics to be prescribed. Second, probiotic consumption has been shown to reduce duration of symptoms.^12^ If an infection resolves more quickly, there is a good probability that a person will seek less medical care, reducing the opportunity for an antibiotic to be prescribed. Third, probiotic consumption may simply be a replacement for antibiotics as patients and clinicians manage self-limited illnesses. Indeed, much antibiotic prescribing for common acute infections is likely unnecessary and may be a response to social and emotional factors,^41–44^ not medical ones. For example, a meta-analysis designed to engage children in addition to parents was effective in decreasing prescribing by 13–40%,^45^ while a systematic review found parent education with clinician behaviour change decreased antibiotic prescribing rates by 6–21%.^46^ This is compared to the 29% decrease we observed among infants in children. The recommendation to take a probiotic may offer an intervention that can fulfil the need to “do something”.

Antibiotic overuse contributes to antibiotic resistance.^16–18^ Probiotic consumption may directly and indirectly reduce such a burden. Our results suggest that further research in this area may be important. If studies that specifically evaluate the impact of probiotic consumption on antibiotic use consistently demonstrate an impact, the potential public health implications could be significant.

## Summary and conclusions

Infants and children who are given probiotics (*Lactobacillus* and *Bifidobacterium* strains) to reduce the risk for acute respiratory tract infections and acute lower digestive tract infections, have a statistically significantly lower relative risk of being prescribed antibiotics (Pooled Relative Risk = 0.71, 95% CI: 0.54–0.94). When restricted to studies with a low risk of bias, the pooled relative risk was 0.46 (95% CI: 0.23–0.97). Significant statistical heterogeneity was present in effect size estimates, which appeared to be due to one outlier.

Probiotics, provided to reduce the risk for common acute infections in infants and children, may be associated with reduced antibiotic use. Additional well-designed studies, preferably RCTs of healthy individuals, are needed to substantiate these findings in children and explore similar findings in other population groups.

## Funding and conflicts of interest

All authors received compensation for travel and lodging by International Scientific Association for Probiotics and Prebiotics (ISAPP) to attend the 2016 Annual ISAPP Meeting in Turku, Finland where the manuscript was conceived. SK received an honorarium from ISAPP as partial compensation for time spent conducting this systematic review. She reports no other conflicts of interest. DT reports receiving consultation fees from Pfizer Consumer Health for providing statistical education. In addition, he has received research and travel support from ISAPP, a non-profit scientific society. ILW is employed by the Danone Company. MES consults with numerous companies engaged in probiotic business, but does not have any financial stake in any company. She serves on scientific advisory boards for Danone, Yakult, The Dannon Company, Clorox and Winclove. ALS has received support for travel and lodging from ISAPP, to participate in research conferences and meetings sponsored by ISAPP. DM has been an expert for a probiotic product for Bayer and Pharmative and has received support for travel and lodging from ISAPP, to participate in research conferences and meetings sponsored by ISAPP. KG, HV, GC and JAL state no conflicts of interest.

**Key points**

- Evidence suggests that probiotic supplementation reduces episodes of common infectious diseases including respiratory tract infections and diarrhea
- Infants and children given probiotics to avoid or reduce the risk for certain infections have a 29% lower relative risk of being prescribed antibiotics. For patients with a 25% risk of being prescribed, the number needed to treat to avoid one prescription is 13.8.
- Probiotic consumption may be a replacement for antibiotics as patients and clinicians manage self-limited illnesses

## Supplementary Material

cky185_Supp

## Notes

PROSPERO Registration Number: CRD42016052694
