# Supplementary material for: Does probiotic consumption reduce antibiotic utilization for common acute infections? A systematic review and meta-analysis
Source: Eur J Public Health. 2018 Nov 14;29(3):494–9. doi: 10.1093/eurpub/cky185 (PMC6532828; doi:10.1093/eurpub/cky185)
Supplement: cky185_Supp [file cky185_supp.zip › cky185-Suppl_data/cky185_Supplementary_Table1.docx]

Supplementary Table I: Characteristics of included studies

| **Reference, study country, study design, condition(s) evaluated** | **Aim(s) of study, population, baseline characteristics** | **Intervention (number randomized)** | **Control (number randomized)**  **duration of intervention** | **Antibiotic utilization (as presented by the study authors)** |
| --- | --- | --- | --- | --- |
| Allen et al. 2010 UK  Randomized, double-blind, placebo-controlled trial  The authors evaluated a number of ‘common’ conditions including upper and lower respiratory tract infections | Aim(s): To evaluate the safety of a bacterial dietary supplement for the prevention of atopy in infants.  Mother-infant dyads attending clinics were recruited  Baseline characteristics of the infants were not reported except for birthweight which was 3.49 kg (2.1 to 4.9) in the treatment group and 3.55 kg (2.0 to 5.2) in the placebo group | Given to women during last month of pregnancy and to their infants from birth to 6 months:  Capsules containing  *Lactobacillus salivarius* CUL61 6.25 x 10^9^ colony-forming units (cfu), *Lactobacillus paracasei* CUL08 1.25 x 10^9^ cfu, *Bifidobacterium animalis subsp. lactis* CUL34 1.25 x 10^9^ cfu, and *Bifidobacterium bifidum* CUL20 1.25 x 10^9^ cfu (n=220)  Capsules were mixed with formula or expressed breast milk or sprinkled directly into the baby’s mouth | Placebo capsules containing maltodextrin (n=234)  Duration of intervention: Up to 28 weeks | 39/192 (20.3%) infants in treatment group and 48/195 (24.6%) infants in placebo group received antibiotics (recorded between ages of 9 to 28 weeks) (data for 327 infants also recorded by study authors for 0 to 8 weeks but were not included in this review) |
| Cáceres et al. 2010  Chile  Randomized, double-blind, placebo-controlled trial  Acute respiratory infections, and acute otitis media | Aim(s): To evaluate whether the regular intake of the probiotic *Lactobacillus rhamnosus* HN001 affects the frequency, severity or duration of ARI in children attending day care centres in the winter season.  Children aged 1-5 years attending 4 day care centres  Male (%): 47% in probiotic group and 49% in placebo group;  Mean age: 3.1 (SD 1.1) years in probiotic group and 3.2 (SD 1.0) years in placebo group | Milk-based product containing *Lactobacillus rhamnosus* HN001 10^8^ cfu (n=203)  Each child received one bottle per day from Monday to Friday | Placebo (n=195)  Duration of intervention: 3 months | Number of days with antibiotics:  Probiotic group: 1.54 days (SD 3.64) (n=203)  Placebo group: 1.45 (SD 3.55) (n=195) |
| Gerasimov et al. 2016  Ukraine  Randomized, double-blind, placebo-controlled trial  Acute respiratory infections | Aim(s): To assess the role of the short term use of probiotics in prevention/modification of ARI in children at an increased risk of infection due to extended indoor exposure to a sick household.  Healthy children aged 3 to 12 years (at risk of ARI)  Male (%): 54% in probiotic group and 55% in placebo group; Median age: 7 years (IQR 5 to 10) in probiotic group and 6 years (IQR 5 to 10) in placebo group;  Common colds in the past 12 months: 96/113 (85%) in probiotic group and 89/112 (79%) in placebo group; Episodes per child: median 2 (IQR 1 to 4) in probiotic group and median 2 (IQR 2 to 3) in placebo group. | A mixture of *Lactobacillus acidophilus* DDS-1 and *Bifidobacterium lactis* UABLA-12 (in a proportion of 1:4 by cell count with fructooligosaccharide (FOS) in rice maltodextrin. A dose of the supplement, measured as a quarter of a teaspoon or ∼1 g of powder, contained 5 billion colony-forming units (CFUs) (n=120)  A dose of the probiotic or control powder was reconstituted in 25-50 ml of tepid water or juice immediately fed once a day | Placebo containing maltodextrin (n=120)  Duration of intervention: The test supplement was given when an individual with ARI was identified in the dwelling for 14 days or until resolution of secondary ARI in a child | The authors reported that 8 in the probiotic group and 8 in the control group received antibiotic prescriptions |
| Gutierrez-Castrellon et al. 2014  Mexico  Randomized, double-blind, placebo-controlled trial  Diarrhea | Aim(s): To evaluate the efficacy of prophylactic use of  *L reuteri* DSM 17938 in reducing the frequency and duration of diarrheal episodes and other health outcomes in Mexican children in the day care setting and further, to assess cost-effectiveness of the intervention.  Children attending 4 day care centres  Male (%): 52% in probiotic group and 53% in placebo group; Mean age: 20.6 (SD 4.1) months in probiotic group and 21.1 (SD 4.2) months in placebo group | *Lactobacillus reuteri* DSM 17938 at  a dose of 1 x10^8^ cfu (n=168) 5 drops (∼0.2 mL) of an oil formulation from a dropper bottle was given to the children directly in the mouth, during the first feed of the day in the home (once per day) | Placebo (n=168)  Duration of intervention: 12 weeks | The number of days of antibiotic use was 2.7 (SD 0.9) days in the probiotic group (n=168) and 4.1 (SD 1.3) days in the placebo group (n=168) during the 12 week treatment period |
| Hatakka et al. 2007  Finland  Randomized, double-blind, placebo-controlled trial  Upper respiratory infection, and acute otitis media | Aim(s): To examine whether probiotics would reduce the occurrence or duration of acute otitis media (AOM), or the nasopharyngeal carriage of otitis pathogens in otitis-prone children.  Otitis-prone children (10 months to 6 years)  Male (%): 57% in probiotic group and 58% in placebo group; Mean age: 2.4 years (range 0.8 to 6.0) in probiotic group and 2.4 years (range 0.9 to 5.6) in placebo group;  Median number of antimicrobial treatments during preceding 12 months: 6 (range 1 to 16) in the probiotics group and 6 (3 to 13) in the placebo group; Respiratory infections during lifetime (probiotics vs. placebo): acute respiratory infection: 98% vs. 99%; bronchitis: 32% vs. 29%; pneumonia: 5% vs. 7%; sinusitis: 3% vs. 4%; laryngitis: 24% vs. 21%; tonsilitis: 8% vs. 6%; Tympanostomy: 12% vs. 15%; Adenoidectomy: 32% vs. 28% | One gelatin capsule per day containing a combination of probiotic bacteria (*Lactobacillus rhamnosus* GG, ATCC 53103; *L. rhamnosus* LC 705; *Bifidobacterium breve* 99; *Propionibacterium freudenreichii* ssp *shermanii* JS, 8–9 x10^9^ cfu/capsule of each strain (n=155)  Powder into milk or a milk product was given, or alternatively as a capsule if the child was able to swallow it | Placebo capsule containing cellulose mcrocrystalline (n=154)  Duration of intervention: 6 months | The number of antimicrobial treatments during the 6-month intervention was median 1 (IQR 1 to 3) in the probiotic group (n=135) and median 1 (0 to 2) in the placebo group (n=134) (p value not reported) |
| Hatakka et al. 2001  Finland  Randomized, double-blind, placebo-controlled trial  Acute respiratory and gastrointestinal infections, and acute otitis media | Aim(s): To examine whether long term consumption of a probiotic milk could reduce gastrointestinal and respiratory infections in children in day care centres.  Children aged 1-6 years attending one of 18 day care centres  Male (%): 54% in probiotic group and 48% in placebo group;  Mean age: 4.6 years (range 1.3 to 6.8) in probiotic group and 4.4 years (range 1.3 to 6.7) in placebo group;  Health in past 12 months:  Respiratory infections (probiotic group vs. placebo group respectively) 0-2: 50% vs. 47%; 3-4: 36% vs. 30%; >5: 14% vs. 23%  Gastrointestinal infections 0-1: 79% vs, 75%; >2: 21% vs. 25%  Antibiotic treatments 0-1: 65% vs. 64%; >2: 35% vs. 36% | Milk containing *Lactobacillus rhamnosus* GG (5-10 x 10^5^ cfu/ml) 3 times a day, 5 days a week (n=296) | Placebo milk without probiotic 3 times a day, 5 days a week (n=298)  Duration of intervention: 7 months | Number of children with at least one course of antibiotics:  119/252 (47%) in the probiotics group and 144/261 (55%) in the placebo group aOR 0.78 (95% CI: 0.54 to 1.11, p=0.17).  For respiratory tract infections only: 111/252 (44%) in the probiotics group and 140/261 (54%) in the placebo group aOR 0.72 (95% CI: 0.50 to 1.03, p=0.08). |
| Hojsak et al. 2010a  Croatia  Randomized, double-blind, placebo-controlled trial  Acute respiratory and gastrointestinal infections | Aim(s): To investigate the role of *Lactobacillus rhamnosus* GG in the prevention of gastrointestinal and respiratory tract infections in children who attend day care centres.  Children aged 1-7 years attending day care centres  Male (%): 56.1% in probiotic group and 55.6% in placebo group;  Mean age: 51.9 months (range 13 to 86) in probiotic group and 53.6 months (range 13 to 83) in placebo group (only height and weight also reported) | Fermented milk product (100 ml) containing *Lactobacillus rhamnosus* GG (10^9^ cfu) daily (n=139) | Placebo fermented milk product (100 ml) without probiotic, daily (n=142)  Duration of intervention: 3 months | Number of children treated with antibiotics (bacterial cause): 23/139 (16.5%) in the probiotics group and 34/142 (23.9%) in the placebo group (p=0.12)  The children were treated with antibiotics due to: acute otitis media (8 children in LGG group vs. 13 in placebo group, p=0.28); pharyngitis (10 children in LGG group vs. 14 in placebo group, p=0.43); purulent rhinitis (4 children in LGG group vs. 6 in placebo group, p=0.54) and pneumonia (one child in both groups). |
| Hojsak et al. 2010b  Croatia  Randomized, double-blind, placebo-controlled trial  Acute respiratory and gastrointestinal infections | Aim(s): To evaluate the role of Lactobacillus GG in the prevention of nosocomial gastrointestinal and respiratory tract infections in a pediatric hospital setting.  Children with a mean age of 10.2 years (SD 5.1) attending a hospital  Male (%): 50.8% in probiotic group and 55.7% in placebo group; Mean age: 9.9 years (SD 5.1) in probiotic group and 10.6 years (SD 5.0) in placebo group;  Non-infectious gastrointestinal disorders: 60 (16.0%) in probiotic group and 49 (13.4%) in placebo group;  Urinary tract disorders: 21 (5.6%) in probiotic group and 21 (5.7%) in placebo group;  Noninfectious pulmonary and immunologic disorders: 73 (19.4%) in probiotic group and 70 (19.1%) in placebo group | Fermented milk product (100 ml) containing *Lactobacillus rhamnosus* GG (10^9^ cfu) daily (n=376) | Placebo fermented milk product (100 ml) without probiotic, daily (n=366)  Duration of intervention: Median 5 days (range 3 to 7 in probiotics group) and median 4 days (range 4 to 6 days) in placebo group | Number of children treated with antibiotics (a bacterial cause was determined in 5 patients with upper respiratory tract infections):  1/376 (0.3%) in the probiotics group and 4/366 (1.1%) in the placebo group (p value not reported). No patients with gastrointestinal infections required antibiotic treatment  . |
| Kumpu et al. 2012  Finland  Randomized, double-blind, placebo-controlled trial  Acute respiratory and gastrointestinal infections, and acute otitis media | Aim(s): To assess whether long-term daily consumption of milk containing probiotic *Lactobacillus rhamnosus* GG decreases respiratory illness in children.  Children aged 2-6 years attending one of 60 day care centres  (Authors reported info only on those analysed: GG group (n=251) and placebo group (n=250))  Male (%): 53% in both groups;  Mean age: 4.0 years (SD 1.3) in GG group and 4.0 years (SD 1.4) in placebo group;  Median number of respiratory infections*: 4 (IQR 2, 6) in both groups;  Median number of otitis media*: 1 (IQR 0, 1) in GG group and 1 (IQR 0, 2) in placebo group;  Antibiotic treatments*: 1 (IQR 0, 2) in both groups  *during the past 12 months (as reported by parents) | Milk containing *Lactobacillus rhamnosus* GG (amounts ranged from 2.0 x 10^5^ to 1.9 x 10^6^ cfu/ml) 3 times a day (n=261) | Placebo milk without probiotic 3 times a day (n=262)  Duration of intervention: 28 weeks | ‘Percentage of children prescribed antibiotics at least once during the intervention’: 89/251 (35%) in the GG group and 86/250 (34%) in the placebo group (p=0.80), with the median number of treatments as 1 (IQR 1, 2) (data not reported by group). |
| Leyer et al. 2009  China  Randomized, double-blind, placebo-controlled trial  Acute respiratory and gastrointestinal infections | Aim(s): To investigate whether the daily consumption of *Lactobacillus acidophilus* NCFM or a combination of *L acidophilus* NCFM and *Bifidobacterium animalis* subsp *lactis* Bi-07 would affect the incidence and duration of fever, rhinorrhea, and cough and the incidence of antibiotic prescriptions among otherwise healthy children.  Children aged 3-5 years attending a day care centre  Male (%): 42.0% in single probiotic group, 48.2% in combined probiotic group and 42.3% in placebo group;  Mean age: 3.7 years (SD 0.7) in single probiotic group, 3.8 years (SD 0.6) in combined probiotic group and 4.1 years (SD 0.54) in placebo group (only weight also reported) | Intervention 1: Probiotic sachet (added to milk) containing *Lactobacillus acidophilus* NCFM (5.0 x 10^9^ cfu/g) twice per day (daily dose 1.0 x 10^10^ cfu) (n=110)  Intervention 2: Probiotic sachet (added to milk) containing *Lactobacillus acidophilus* NCFM and *B animalis* subsp *lactis* Bi-07 for a daily dose 1.0 x 10^10^ cfu (n=112) | Placebo sachet (sucrose), daily (n=104)  Duration of intervention: 6 months | ‘Incidence of antibiotic use’:  18/110 (16.4%) in *Lactobacillus acidophilus* group; 9/112 (8.0%) in the L acidophilus/ *Bifidobacterium lactis* group and 57/104 (54.8%) in the placebo group (p=0.0002 single vs. placebo and p<0.0001 combined vs. placebo) |
| Merenstein et al. 2010  USA  Randomized, double-blind, placebo-controlled trial (cluster randomized by households)  Acute respiratory and gastrointestinal infections, and acute otitis media | Aim(s): To evaluate whether a fermented dairy drink containing the probiotic strain *Lactobacillus casei* DN-114 001 could reduce the incidence of common infectious diseases (CIDs) and the change of behavior because of illness in children.  Healthy children aged 3-6 years attending day care/schools  Male (%): 50% in probiotic group and 53.1% in placebo group; Mean age: 4.9 years (SD 1.1) in probiotic group and 4.9 years (SD 1.1) in placebo group; Overall health (rated on a scale of 1-10, with higher scores indicating better health) were 9.2 (SD 0.9) in both the probiotic and placebo groups | Strawberry-flavored DanActive, a fermented probiotic dairy drink, ‘Actimel’ containing *L. casei* DN-114 001/  CNCM I-1518 (also named *Lactobacillus paracasei* subsp. *paracasei* after the current nomenclature) ( 1 x 10^8^ cfu/g) combined with two cultures commonly used in yogurt, *Streptococcus thermophiles* and Lactobacillus bulgaricus (>10^7^ cfu/g) (n=314) | Sweetened, flavoured non-fermented acidified drink (n=324)  Duration of intervention: 90 days | The authors reported a difference in antibiotics (n=58 in the intervention and n=69 for control, p=0.002). |
| Rautava et al. 2009  Finland  Randomized, double-blind, placebo-controlled trial  Acute respiratory and gastrointestinal infections, and otitis media | Aim(s): To assess whether probiotics might be effective in reducing the risk of infections in infancy.  Full-term infants requiring formula before 2 months of age, recruited from community and well-baby clinic  Inclusion/exclusion criteria: Infants requiring formula before 2 months of age. Infants with chronic disease were excluded.  Male (%): 50% in probiotics group and 48% in placebo group; Mean age at start of intervention: 38 days (range 6 to 65) in probiotic group and 35 days (range 2 to 59) in placebo group (Authors reported info only on those analysed: probiotics group (n=32) and placebo group (n=40)) | Formula supplemented with *Lactobacillus rhamnosus* GG and *Bifidobacterium lactis* BB-12 (1 x 10^10^ cfu daily of both LGG and BB-12) (n=38) | Placebo (microcrystalline cellulose) (n=43)  Duration of intervention: Until one year of age | ‘Antibiotic use’ during the first seven months of life: 10/32 (31%) in the probiotics group and 24/40 (60%) in the placebo group, RR 0.52 (95% CI: 0.29 to 0.92), p=0.015. |
| Rerksuppaphol and Rerksuppaphol 2012  Thailand  Randomized, double-blind, placebo- controlled trial  Acute respiratory infections | Aim(s): To assess the efficacy of a two-strain combination probiotic for prevention of common cold symptoms in healthy school children.  Healthy children aged 8-13 years attending a public school in a rural area  Male (%): 38% in probiotic group and 48% in placebo group; Mean age: 12 years (SD 0.6) in probiotic group and 11 years (SD 0.9) in placebo group | *Lactobacillus acidophilus* (min of 10^9^ capsule) and *Bifidobacterium bifidum* (min 10^9^ capsule) twice a day (n=40) | Oral rehydration salts powder in capsules (n=40)  Duration of intervention: 3 months | ‘Antibiotic use’: 2/40 (5%) in the probiotics group and 5/40 (13%) in the placebo group, p=0.432 |
| Ringel-Kulka et al. 2015  USA  Randomized, double-blind, placebo- controlled trial  ‘Fever, diarrhea, or other illnesses’ | Aim(s): To assess the effects of daily consumption of a synbiotic yogurt drink on the health, growth, and quality of life of healthy children 12-48 months of age in out-of-home child care  Healthy children aged 12 to 48 months attending one of 29 child care centers  Male (%): 51.3% in probiotic group and 43.8% in placebo group; Mean age: 2.5 years (SD not reported) in probiotic group and 2.4 years (SD not reported) in placebo group; Breastfed: 73.7% in probiotic group and 67.1% in control | 91mL/97 g synbiotic yogurt drink containing 2 probiotic yogurt starters, *Streptococcuthermophilus* and *Lactobacillus bulgaricus* (1 x10^8^ cfu/g), the probiotic *Bifidobacterium animalis* subspecies *lactis* (BB-12) (5 x10^9^ cfu/serving), and 1 g of inulin (n=82)  One bottle of the dairy drink was consumed each day | Acidified, flavored milk, without bacteria starters, probiotics, or inulin (n=85)  Duration of intervention: 16 weeks | In the modified intention-to-treat analysis, 25 of 76 patients in the probiotic group received an antibiotic versus 27 of 73 patients in the control group |
| Taipale et al. 2011  Finland  Randomized, double-blind, placebo- controlled trial  Acute respiratory and gastrointestinal infections, and otitis media | Aim(s): To assess if the administration of BB-12 to young healthy children could reduce the risk of acute infectious diseases, including AOM and respiratory infections.  Healthy newborn infants  Male (%): 50% in probiotic group and 57% in control group; probiotic vs. control: gestational age: mean 40 months (SD 1.1) vs. 39.7 months (SD 1.2); exclusive breastfeeding: 3.4 months (SD 1.7) vs. 3.8 (SD 1.9); total breastfeeding: 7.1 months (SD 2.3) vs. 6.9 months (SD 3.1) | *Bifidobacterium animalis* subsp. *lactis* BB-12 (BB-12) (DSM 15 954). Each tablet contained 5 billion cfu plus xylitol (n=55)  Two tablets per day via slow-release pacifier | Xylitol (n=54)  Duration of intervention: 8 months | The use of antibiotics was reported as 10/34 (29%) in the probiotic group and 8/35 (23%) in the control group with a RR of 1.29 (95% CI 0.58 to 2.87), p=0.535  The authors did not explicitly state whether the use of antibiotics was a primary or secondary outcome |
| Weizman et al. 2005  Israel  Randomized, double-blind, placebo- controlled trial  Acute respiratory and gastrointestinal infections, and otitis media | Aim(s): To investigate the effect of 2 different species of probiotics in preventing infections in infants attending child care centers.  Infants (4 to 10 months of age) attending one of 14 child care centers (not breast fed)  Male (%): 47% in BB-12 group, 49% in the L*. reuteri* group and 48% in control group; Age at study entry: 6.9 months (SD 1.6) in BB-12 group, 6.8 (SD 1.7) in the L*. reuteri* group and 6.7 (SD 1.5) in control group; birthweight (percentiled): 42.6 (SD 27.1) in BB-12 group, 47.8 (SD 31.6) in the L*. reuteri* group and 45.2 (SD 28.4) in control group; previous breastfeeding: 73% in BB-12 group, 76% in the L*. reuteri* group and 84% in control group | Intervention 1: Formula supplemented with *Bifidobacterium lactis* (BB-12) 1 x 10^7^ cfu per g of formula powder (n=73)  Intervention 2: Formula supplemented with *Lactobacillus reuteri* (SD 2112) 1 x 10^7^ cfu per g of formula powder (N=68) | Control: Same formula with no supplement of probiotics (n=60)  Infants were fed formula in the child care center and at home.  Duration: 12 weeks | Prescriptions of antibiotics mean (95% CIs): 0.21 (0.12 to 0.30) in BB-12 group (n=73), 0.06 (0.01 to 0.12) in the L*. reuteri* group (n=68) and 0.19 (0.09 to 0.29) in control group (n=60), p=0.037 (*L. reuteri* vs. BB-12 and control).  The indications for antibiotic therapy were otitis media, pneumonia, and upper respiratory infection, and they did not differ among the groups |
| West et al. 2008  Sweden  Randomized, double-blind, placebo- controlled trial  Acute respiratory and gastrointestinal infections, and otitis media | Aim(s): To investigate  the impact of feeding healthy, term infants LF19 during weaning on the number of days with infectious symptoms, antibiotic prescriptions and IgG antibody responses to the routine vaccines against *Haemophilus influenzae* type b (Hib), diphtheria and tetanus.  Healthy infants (4 to 13 months of age)  Male (%): 39% in probiotics group and 45% in control group; birthweight: 3725 grams (SD 516) in probiotics group and 3632 g (SD 503) in control group  There was no difference in the rate of breastfeeding between the groups. Infants were exclusively breastfed for a mean duration of 3.7 (1.4 SD) months and 3.8 (1.1 SD) months in the probiotic and placebo groups respectively (p > 0.05). The mean total duration of breastfeeding was 7.8 (3.3 SD months in both groups | Cereals supplemented with *Lactobacillus paracasei* ssp. *paracasei* strain F19 (LF19); 1 x 10^8^ cfu (one serving of cereals daily) (n=89) | Cereals without LF19 (n=90)  Duration: 9 months | Mean days with antibiotic prescriptions were 1.6 (95% CI 1.3 to 1.9) in the probiotic group (n=84) and 2.2 (95% CI 1.7 to 2.7) in the control group (n=87), p=0.044. |
