# Supplementary material for: Does probiotic consumption reduce antibiotic utilization for common acute infections? A systematic review and meta-analysis
Source: Eur J Public Health. 2018 Nov 14;29(3):494–9. doi: 10.1093/eurpub/cky185 (PMC6532828; doi:10.1093/eurpub/cky185)
Supplement: cky185_Supp [file cky185_supp.zip › cky185-Suppl_data/cky185_Supplementary_Table2.docx]

Supplementary Table II: Risk of bias of the included studies

| **Reference** | **Was randomization carried out appropriately?** | **Was the concealment of treatment allocation adequate?** | **Were the groups similar at the outset of the study in terms of prognostic factors (e.g. previous history of illness that might influence antibiotic use)?** | **Were the care providers, participants and outcome assessors blind to treatment allocation? If any of these were not blinded, what might be the likely impact on the risk of bias (for each outcome)?** | **Were there any unexpected imbalances in drop-outs between groups? If so, were they explained or adjusted for?** | **Is there any evidence to suggest that the authors measured more outcomes than they reported?** | **Did the analysis *of antibiotic use* include an intention-to-treat analysis? If so, were appropriate methods used to account for missing data?** | **Overall risk of bias assessment** |
| --- | --- | --- | --- | --- | --- | --- | --- | --- |
| Allen et al. 2010 | Low | Low | Unclear | Low? | Low | Low | High | High |
| Cáceres et al. 2010 | Low | Unclear | Unclear | Low | High | Unclear | Low | Unclear |
| Gerasimov et al. 2016 | Low | Low | Low | Low | Low | Low | High | High |
| Gutierrez-Castrellon et al. 2014 | Low | Low | Unclear | Low | Low | Low | Low | Low |
| Hatakka et al. 2007 | Low | Low* | Low | Low | Low | Low | High | High |
| Hatakka et al. 2001 | Low | Low* | High | Low | Low | Low | High | High |
| Hojsak et al. 2010a | Low | Low* | Unclear | Low | Low | Low | Low | Low |
| Hojsak et al. 2010b | Low | Low* | Unclear | Low | Low | Low | Low | Low |
| Kumpu et al. 2012 | Low | Low* | Unclear | Low? | Low | Low | High | High |
| Leyer et al. 2009 | Low | Low* | Unclear | Low? | Low | Low | Low | Low |
| Merenstein et al. 2010 | Low | Low | Low | Low | Low | Low | Low | Low |
| Rautava et al. 2009 | Low | Low | Unclear | Low | High | Low | High | High |
| Rerksuppaphol 2012 | Low | Unclear | Unclear | Low | Low | Low | Low | Unclear |
| Ringel-Kulka et al. 2015 | Low* | Low* | Unclear | Low | Low | Low | High | High |
| Taipale et al. 2016 | Low | Low* | Unclear | Low | Low | Unclear | High | High |
| Weizman et al. 2005 | Low | Unclear | Unclear | Low? | Low | Low | Low | Unclear |
| West et al. 2008 | Unclear | Unclear | Unclear | Low | Low | Low | Low? | Unclear |

*Information was obtained from study authors
