# Supplementary material for: Does probiotic consumption reduce antibiotic utilization for common acute infections? A systematic review and meta-analysis
Source: Eur J Public Health. 2018 Nov 14;29(3):494–9. doi: 10.1093/eurpub/cky185 (PMC6532828; doi:10.1093/eurpub/cky185)
Supplement: cky185_Supp [file cky185_supp.zip › cky185-Suppl_data/cky185_Supplementary_Appendix.docx]

**Supplementary Appendix A: Search Strategy**

Ovid MEDLINE and Cochrane Central Register of Controlled Trials (CENTRAL):

1. exp Probiotics/

2. probiotics.mp.

3. 1 or 2

4. exp Antimicrobial Cationic Peptides/

5. antimicrobials.mp.

6. 4 or 5

7. exp Cultured Milk Products/

8. (yogurt or yoghurt).mp.

9. fermented milk produc*.mp.

10. cultured milk produc*.mp.

11. 7 or 8 or 9 or 10

12. exp Lactobacillus/ or Lactobacillus.mp.

13. Bifidobacterium.mp. or exp Bifidobacterium/

14. Saccharomyces cerevisiae/ or Saccharomyces.mp. or Saccharomyces/

15. brewer's yeast.mp.

16. baker's yeast.mp.

17. 14 or 15 or 16

18. Bacillus.mp. or exp Bacillus/

19. Streptococcus thermophilus.mp. or exp Streptococcus thermophilus/

20. exp Propionibacterium/ or Propionibacterium.mp.

21. Lactococcus.mp. or exp Lactococcus lactis/

22. Escherichia coli.mp. or exp Escherichia coli/

23. e coli.mp.

24. 22 or 23

25. Enterococcus.mp. or exp Enterococcus/

26. 3 or 6 or 11 or 12 or 13 or 17 or 18 or 19 or 20 or 21 or 24 or 25

27. exp Fever/ or fever.mp.

28. Pharyngitis.mp. or exp Pharyngitis/

29. sore throa*.mp.

30. 28 or 29

31. Arthralgia.mp. or exp Arthralgia/

32. joint pai*.mp.

33. 31 or 32

34. exp Myalgia/ or Myalgia.mp.

35. muscle pai*.mp.

36. 34 or 35

37. common cold.mp. or exp Common Cold/

38. head cold.mp.

39. 37 or 38

40. human influenza.mp. or exp Influenza, Human/

41. flu.mp.

42. 40 or 41

43. exp Acute Disease/

44. exp Sinusitis/ or Sinusitis.mp.

45. 43 and 44

46. acute Sinusitis.mp.

47. 45 or 46

48. exp Bronchitis/ or bronchitis.mp.

49. 43 and 48

50. acute bronchitis.mp.

51. 49 or 50

52. exp Pneumonia/ or pneumonia.mp.

53. 43 and 52

54. acute pneumonia.mp.

55. 53 or 54

56. exp Peritonitis/ or peritonitis.mp.

57. 43 and 56

58. acute peritonitis.mp.

59. 57 or 58

60. otitis media.mp. or exp Otitis Media/

61. 43 and 60

62. middle ear inflammatio*.mp.

63. middle ear infectio*.mp.

64. 62 or 63

65. 43 and 64

66. acute middle ear inflammatio*.mp.

67. acute middle ear infectio*.mp.

68. 61 or 65 or 66 or 67

69. exp Gastrointestinal Diseases/

70. gastrointestinal diseas*.mp.

71. gastrointestinal infectio*.mp.

72. gastrointestinal illnes*.mp.

73. 69 or 70 or 71 or 72

74. 43 and 73

75. acute gastrointestinal diseas*.mp.

76. acute gastrointestinal infectio*.mp.

77. acute gastrointestinal illnes*.mp.

78. 74 or 75 or 76 or 77

79. exp Diarrhea/ or Diarrhea.mp.

80. diarrhe*.mp.

81. diarrhoe*.mp.

82. 79 or 80 or 81

83. exp Nausea/ or nausea.mp.

84. exp Vomiting/ or vomiting.mp.

85. emes*.mp.

86. 84 or 85

87. exp Respiratory Tract Infections/

88. respiratory tract infectio*.mp.

89. respiratory illnes*.mp.

90. respiratory infectio*.mp.

91. 87 or 88 or 89 or 90

92. 43 and 91

93. acute respiratory tract infectio*.mp.

94. acute respiratory illnes*.mp.

95. acute respiratory infectio*.mp.

96. 92 or 93 or 94 or 95

97. 27 or 30 or 33 or 36 or 39 or 42 or 47 or 51 or 55 or 59 or 68 or 78 or 82 or 83 or 86 or 96

98. 26 and 97

99. antibiotics.mp. or exp *Anti-Bacterial Agents/

100. prebiotics.mp. or exp *Prebiotics/

101. vitamins.mp. or exp *Vitamins/

102. 99 or 100 or 101

103. 98 not 102

104. exp Placebos/

105. placeb*.mp.

106. no treatment.mp.

107. non-treatment.mp.

108. watchful waiting.mp. or exp Watchful Waiting/

109. 104 or 105 or 106 or 107 or 108

110. 103 and 109

111. limit 110 to randomized controlled trial

112. 98 and 109

113. limit 112 to randomized controlled trial

Ovid Embase

1. exp probiotic agent/

2. probiotics.mp.

3. 1 or 2

4. dairy product/ and fermented product/

5. fermented milk produc*.mp.

6. Cultured Milk Produc*.mp.

7. yogurt.mp. or exp yoghurt/

8. 4 or 5 or 6 or 7

9. Lactobacillus.mp. or exp Lactobacillus/

10. Bifidobacterium.mp. or exp Bifidobacterium/

11. exp Saccharomyces/ or Saccharomyces.mp.

12. brewer's yeast.mp.

13. baker's yeast.mp.

14. 11 or 12 or 13

15. Bacillus.mp. or exp Bacillus/

16. Streptococcus thermophilus.mp. or exp Streptococcus thermophilus/

17. Propionibacterium.mp. or exp Propionibacterium/

18. exp Lactococcus/ or Lactococcus.mp.

19. Escherichia coli.mp. or exp Escherichia coli/

20. e coli.mp.

21. e-coli.mp.

22. 19 or 20 or 21

23. exp Enterococcus/ or Enterococcus.mp.

24. 3 or 8 or 9 or 10 or 14 or 15 or 16 or 17 or 18 or 22 or 23

25. exp fever/ or fever.mp.

26. Pharyngitis.mp. or exp pharyngitis/

27. sore throa*.mp.

28. 26 or 27

29. Arthralgia.mp. or exp arthralgia/

30. joint pai*.mp.

31. 29 or 30

32. exp myalgia/ or Myalgia.mp.

33. muscle pai*.mp.

34. common cold.mp. or exp common cold/

35. head cold.mp.

36. 34 or 35

37. exp influenza/ or influenza.mp.

38. human influenza.mp.

39. flu.mp.

40. influenza virus.mp. or exp Influenza virus/

41. flu virus.mp.

42. 37 or 38 or 39 or 40 or 41

43. acute Sinusitis.mp. or exp acute sinusitis/

44. exp bronchitis/

45. exp acute disease/

46. acute illness.mp.

47. 45 or 46

48. 44 and 47

49. acute bronchitis.mp.

50. 48 or 49

51. acute pneumonia.mp. or exp lobar pneumonia/

52. pneumonia.mp. or exp pneumonia/

53. 47 and 52

54. 51 or 53

55. exp peritonitis/

56. 47 and 55

57. acute peritonitis.mp.

58. 56 or 57

59. acute otitis media.mp. or exp acute otitis media/

60. acute middle ear inflammatio*.mp.

61. acute middle ear infectio*.mp.

62. acute ear infectio*.mp.

63. ear infection.mp. or exp ear infection/

64. otitis media.mp. or exp otitis media/

65. middle ear inflammatio*.mp.

66. middle ear infectio*.mp.

67. 63 or 64 or 65 or 66

68. 47 and 67

69. 59 or 60 or 61 or 62 or 68

70. gastrointestinal disease.mp. or exp gastrointestinal disease/

71. gastrointestinal diseas*.mp.

72. gastrointestinal infection.mp. or exp gastrointestinal infection/

73. gastrointestinal infectio*.mp.

74. gastrointestinal illnes*.mp.

75. GI diseas*.mp.

76. 70 or 71 or 72 or 73 or 74 or 75

77. 47 and 76

78. acute GI diseas*.mp.

79. acute gastrointestinal diseas*.mp.

80. acute gastrointestinal infection.mp. or exp acute gastroenteritis/

81. acute gastrointestinal infectio*.mp.

82. acute gastrointestinal illnes*.mp.

83. 77 or 78 or 79 or 80 or 81 or 82

84. Diarrhea.mp. or exp diarrhea/

85. diarrhe*.mp.

86. diarrhoe*.mp.

87. 84 or 85 or 86

88. 47 and 87

89. exp acute diarrhea/

90. 88 or 89

91. nausea.mp. or exp nausea/

92. exp vomiting/ or vomiting.mp.

93. emes*.mp.

94. 92 or 93

95. respiratory tract infection.mp. or exp respiratory tract infection/

96. respiratory illnes*.mp.

97. respiratory infection.mp. or exp respiratory tract infection/

98. 95 or 96 or 97

99. 47 and 98

100. acute respiratory tract infectio*.mp.

101. acute respiratory illnes*.mp.

102. acute respiratory infectio*.mp.

103. 99 or 100 or 101 or 102

104. 25 or 28 or 31 or 32 or 33 or 36 or 42 or 43 or 50 or 54 or 58 or 69 or 83 or 87 or 90 or 91 or 94 or 103

105. 24 and 104

106. exp antibiotic agent/

107. antibiotics.mp.

108. exp antiinfective agent/

109. anti-bacterial agen*.mp.

110. 106 or 107 or 108 or 109

111. exp prebiotic agent/

112. prebioti*.mp.

113. pre-biotics.mp.

114. 111 or 112 or 113

115. exp vitamin/

116. vitami*.mp.

117. 115 or 116

118. exp *antibiotic agent/

119. exp *antiinfective agent/

120. prebiotic agent.mp. or exp *prebiotic agent/

121. exp *vitamin/

122. 112 or 113 or 120

123. 116 or 121

124. 107 or 109 or 118 or 119

125. 122 or 123 or 124

126. 105 not 125

127. limit 126 to randomized controlled trial

128. 110 or 114 or 117

129. 105 not 128

130. limit 129 to randomized controlled trial

131. exp placebo/ or placebo.mp.

132. no treatment.mp.

133. non-treatment.mp.

134. exp watchful waiting/

135. 131 or 132 or 133 or 134

136. 126 and 135

137. limit 136 to randomized controlled trial

Cochrane Database of Systematic Reviews (CDSR) and Database of Abstracts of Reviews of Effects (DARE):

1. probiotics.mp. [mp=title, short title, abstract, full text, keywords, caption text]

2. fermented milk produc*.mp.

3. cultured milk produc*.mp.

4. Lactobacillus.mp.

5. Bifidobacterium.mp.

6. Saccharomyces.mp.

7. brewer's yeast.mp.

8. baker's yeast.mp.

9. Bacillus.mp.

10. Streptococcus thermophilus.mp.

11. Propionibacterium.mp.

12. Lactococcus.mp.

13. Escherichia coli.mp. [mp=title, short title, abstract, full text, keywords, caption text]

14. e coli.mp. [mp=title, short title, abstract, full text, keywords, caption text]

15. e-coli.mp. [mp=title, short title, abstract, full text, keywords, caption text]

16. 13 or 14 or 15

17. Enterococcus.mp. [mp=title, short title, abstract, full text, keywords, caption text]

18. 1 or 2 or 3 or 4 or 5 or 6 or 7 or 8 or 9 or 10 or 11 or 12 or 16 or 17

19. fever.mp. [mp=title, short title, abstract, full text, keywords, caption text]

20. Pharyngitis.mp. [mp=title, short title, abstract, full text, keywords, caption text]

21. sore throa*.mp. [mp=title, short title, abstract, full text, keywords, caption text]

22. 20 or 21

23. Arthralgia.mp. [mp=title, short title, abstract, full text, keywords, caption text]

24. joint pai*.mp. [mp=title, short title, abstract, full text, keywords, caption text]

25. 23 or 24

26. Myalgia.mp. [mp=title, short title, abstract, full text, keywords, caption text]

27. muscle pai*.mp. [mp=title, short title, abstract, full text, keywords, caption text]

28. 26 or 27

29. common cold.mp. [mp=title, short title, abstract, full text, keywords, caption text]

30. head cold.mp. [mp=title, short title, abstract, full text, keywords, caption text]

31. 29 or 30

32. Influenza, Human.mp. [mp=title, short title, abstract, full text, keywords, caption text]

33. human influenza.mp. [mp=title, short title, abstract, full text, keywords, caption text]

34. flu.mp. [mp=title, short title, abstract, full text, keywords, caption text]

35. flu virus.mp. [mp=title, short title, abstract, full text, keywords, caption text]

36. influenza virus.mp. [mp=title, short title, abstract, full text, keywords, caption text]

37. 32 or 33 or 34 or 35 or 36

38. acute Sinusitis.mp. [mp=title, short title, abstract, full text, keywords, caption text]

39. acute bronchitis.mp. [mp=title, short title, abstract, full text, keywords, caption text]

40. acute pneumonia.mp. [mp=title, short title, abstract, full text, keywords, caption text]

41. acute peritonitis.mp. [mp=title, short title, abstract, full text, keywords, caption text]

42. acute otitis media.mp. [mp=title, short title, abstract, full text, keywords, caption text]

43. acute middle ear inflammatio*.mp. [mp=title, short title, abstract, full text, keywords, caption text]

44. acute middle ear infectio*.mp. [mp=title, short title, abstract, full text, keywords, caption text]

45. acute ear infectio*.mp. [mp=title, short title, abstract, full text, keywords, caption text]

46. 42 or 43 or 44 or 45

47. acute GI diseas*.mp. [mp=title, short title, abstract, full text, keywords, caption text]

48. acute gastrointestinal diseas*.mp. [mp=title, short title, abstract, full text, keywords, caption text]

49. acute gastrointestinal infectio*.mp. [mp=title, short title, abstract, full text, keywords, caption text]

50. acute gastrointestinal illnes*.mp. [mp=title, short title, abstract, full text, keywords, caption text]

51. 47 or 48 or 49 or 50

52. Diarrhea.mp. [mp=title, short title, abstract, full text, keywords, caption text]

53. diarrhe*.mp. [mp=title, short title, abstract, full text, keywords, caption text]

54. diarrhoe*.mp. [mp=title, short title, abstract, full text, keywords, caption text]

55. 52 or 53 or 54

56. nausea.mp. [mp=title, short title, abstract, full text, keywords, caption text]

57. vomiting.mp. [mp=title, short title, abstract, full text, keywords, caption text]

58. emes*.mp. [mp=title, short title, abstract, full text, keywords, caption text]

59. 57 or 58

60. acute respiratory tract infectio*.mp. [mp=title, short title, abstract, full text, keywords, caption text]

61. acute respiratory illnes*.mp. [mp=title, short title, abstract, full text, keywords, caption text]

62. acute respiratory infectio*.mp. [mp=title, short title, abstract, full text, keywords, caption text]

63. 60 or 61 or 62

64. 19 or 22 or 25 or 28 or 31 or 37 or 38 or 39 or 40 or 41 or 46 or 51 or 55 or 59 or 63

65. 18 and 64

66. antibiotics.mp. [mp=title, short title, abstract, full text, keywords, caption text]

67. Anti-Bacterial Agents.mp. [mp=title, short title, abstract, full text, keywords, caption text]

68. prebiotics.mp. [mp=title, short title, abstract, full text, keywords, caption text]

69. vitamins.mp. [mp=title, short title, abstract, full text, keywords, caption text]

70. 66 or 67 or 68 or 69

71. 65 not 70

72. placebo.mp.

73. no treatment.mp.

74. non-treatment.mp. [mp=title, short title, abstract, full text, keywords, caption text]

75. Watchful Waiting.mp. [mp=title, short title, abstract, full text, keywords, caption text]

76. 72 or 73 or 74 or 75

77. 71 and 76

102. acute respiratory infectio*.mp.

103. 99 or 100 or 101 or 102

104. 25 or 28 or 31 or 32 or 33 or 36 or 42 or 43 or 50 or 54 or 58 or 69 or 83 or 87 or 90 or 91 or 94 or 103

105. 24 and 104

106. exp antibiotic agent/

107. antibiotics.mp.

108. exp antiinfective agent/

109. anti-bacterial agen*.mp.

110. 106 or 107 or 108 or 109

111. exp prebiotic agent/

112. prebioti*.mp.

113. pre-biotics.mp.

114. 111 or 112 or 113

115. exp vitamin/

116. vitami*.mp.

117. 115 or 116

118. exp *antibiotic agent/

Web of Science - SCI

#40        #39 AND #38

#39        TS= (RCT OR randomized controlled tria* OR randomized control tria*)

#38        #35 NOT #34       Refined by: DOCUMENT TYPES: (ARTICLE) AND [excluding]: DOCUMENT TYPES:
 (PROCEEDINGS PAPER OR BOOK CHAPTER OR RETRACTED PUBLICATION)

#37        #35 NOT #34       Refined by: DOCUMENT TYPES: (ARTICLE)

#36        #35 NOT #34

#35        #30 AND #13

#34        #33 OR #32 OR #31

#33        TS= vitami*

#32        TS= prebioti*

#31        TS= (Anti-Bacterial Agent* OR antibioti* OR antibiotic agen*)

#30        #29 OR #28 OR #27 OR #26 OR #25 OR #24 OR #23 OR #22 OR #21 OR #20 OR #19 OR #18 OR #17 OR
 #16 OR #15 OR #14

#29        TS= (Pharyngitis OR sore throa*)

#28        TS= (acute otitis media OR acute middle ear inflammatio* OR acute middle ear infectio*)

#27        TS= (acute respiratory tract infectio* OR acute respiratory tract diseas* OR acute respiratory illnes*
OR acute respiratory infectio* OR acute respiratory tract illnes* OR acute respiratory diseas*)

#26        TS= (vomiting OR emesis OR emeses)

#25        TS= Nausea

#24        TS= (Diarrhea OR diarrhoea)

#23        TS= (acute Gastrointestinal Disease* OR acute GI disease* OR acute gastrointestinal infection* OR
acute gastrointestinal illness*)

#22        TS= acute peritonitis

#21        TS= acute pneumonia

#20        TS= acute bronchitis

#19        TS= acute sinusitis

#18        TS= (human Influenza OR flu OR influenza virus)

#17        TS= (common cold OR head cold OR human rhinovirus)

#16        TS= (Myalgia OR muscle pain*)

#15        TS= (Arthralgia OR joint pain*)

#14        TOPIC: (fever)

#13        #12 OR #11 OR #10 OR #9 OR #8 OR #7 OR #2 OR #3 OR #6 OR #5 OR #4 OR #1

#12        TOPIC: (Enterococcus)

#11        TS=(Escherichia coli OR e-coli OR e coli)

#10        TOPIC: (Lactococcus)

#9          TOPIC: (Propionibacterium)

#8          TOPIC: (Streptococcus thermophilus)

#7          TOPIC: (Bacillus)

#6          TOPIC: (Saccharomyces) OR TOPIC: (brewer's yeast) OR TOPIC: (baker's yeast)

#5          TOPIC: (Bifidobacterium)

#4          TOPIC: (Lactobacillus)

#3          TS=(yogur* OR yoghur*)

#2          TS=(culture milk produc* OR fermented milk produc*)

#1          TOPIC: (probiotic*)
